# Collagen I-to-III ratio data in healing rabbit Achilles tendon tissue treated with PDGF-BB: Comparison of dual Herovici staining with two single immunohistochemical labelings for Collagen I and Collagen III

**DOI:** 10.1016/j.dib.2026.112864

**Published:** 2026-05-18

**Authors:** Gabriella Meier Bürgisser, Olivera Evrova, Pietro Giovanoli, Maurizio Calcagni, Johanna Buschmann

**Affiliations:** Division of Plastic Surgery and Hand Surgery, University Hospital of Zurich, 8091 Zurich, Switzerland

**Keywords:** Tendon repair, Fibrotic adhesion, Achilles tendon, Tubular implant, Electrospinning, PDGF-BB

## Abstract

After tendon rupture repair, the healing tendon tissue is characterized by an initial, immature deposition of collagen III, which is gradually replaced by mature collagen I. Due to common complications like re-rupture and peritendinous adhesions, a coaxially electrospun DegraPol tube releasing PDGF-BB was developed and tested in a rabbit Achilles tendon full-transection model after pulling it over the conventionally sutured tendon. Beyond cellularity and vascularization, the collagen I-to-III ratio serves as a key indicator of the healing progress. We provide comprehensive data on this ratio at three weeks post surgery, using two methods: Herovici (HV) dual-colour staining and immunohistochemical labelling (IHC) for collagen I and III. Ratios were calculated via colour histograms: red-to-blue for HV, and red-to-green for DAB stained IHC. Data are provided for five distinct anatomical zones: native tendon (NZ), similar-to-native (SZ), reactive zone/interface (RZ), hot zone/core (HZ), and intraspun zone within the fiber mesh pores (IS).

## Graphical abstract

**Figure:**
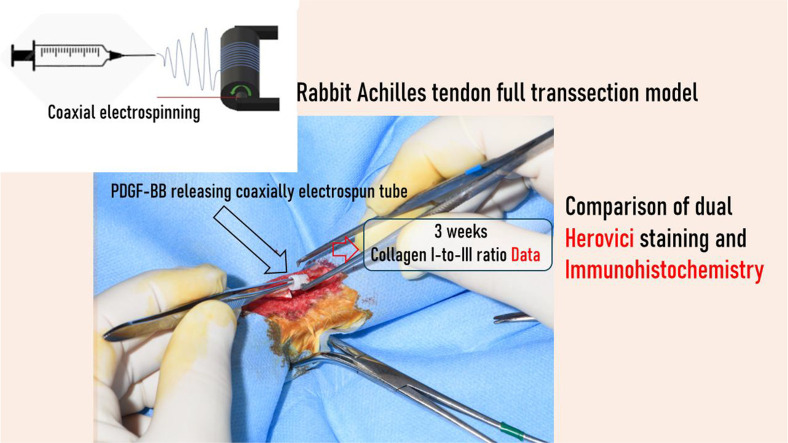

Specifications Table

**Table:** 

Subject	Herovici dual staining of collagen I and III in the healing rabbit Achilles tendon tissue; immunohistochemical staining for Collagen I and III
Specific subject area	rabbit Achilles tendon in vivo model; collagen I-to-III ratio across different anatomical zones
Data format	The data consist of raw and analysed data.
Type of data	Excel file of the type *.xlsx* file
Data collection	Data collection was performed using histological sections of rabbit Achilles tendons treated with pure DegraPol® tubes or with PDGF-BB releasing coaxially electrospun DegraPol® tubes to determine the collagen I-to-III ratio.
Data source location	All data were collected in Zurich, Switzerland, at the University Hospital Zurich in the laboratories of the Department for Plastic Surgery and Hand Surgery.
Data accessibility	Repository name: Mendeley Data Data identification number: 10.17632/n4rcfcr3hj.1 and 10.17632/bhj8vcy58h.1 Direct URL to data: Collagen I-to-III ratio data in healing tendon tissue: comparison of dual Herovici staining with two single immunohistochemical labelings for Collagen I and Collagen III (English version) - Mendeley Data and Spectral Analysis of Herovici stained sections of PDGF-BB DegraPol® or pure DegraPol® tube treated rabbit Achilles tendons and native tendons - Mendeley Data
Related research article	Therapeutically Induced Modulation of Collagen I-to-III Ratio Three Weeks After Rabbit Achilles Tendon Full Transection - PubMed

## 1 Value of the Data

• Our data can be reused, if other growth factors than PDGF-BB are incorporated in tubular delivery tubes (also for other polymers than DegraPol®) across different anatomical zones.
• These data can be reused, if other species than the rabbit Achilles tendon are studied.
• These data can be compared to other connective tissue healing at 3 weeks post-operation, such as ligaments.
• The data can be compared to the rabbit Achilles tendon full transection model at other time points post-operation, for example 6 or 12 weeks, respectively.
• Our collagen I-to-III ratios could be useful for diffusion studies of growth factors with different molecular weights because the distinct anatomical zones exhibit different distances to the drug releasing tubular implant material.
• The spectral analysis data of the histograms may be useful for other assessments than the red-to-blue ratio that was used in the Herovici-based data sets to calculate the collagen I-to-III ratio.

## 2 Background

Connective tissue healing status can be monitored by time- and local-dependent cell density or vascularization, however, the collagen I-to-III ratio represents a good proxy for how advanced the healing has progressed; because initial collagen III deposition is gradually replaced by collagen I, the mature and remaining collagen type [1]. Particularly for tendon healing studies, this offers an easy readout, as histological sections can be stained for example with the dual Herovici staining or by immunohistochemical labeling for collagen I and III, respectively. In a study of tendon rupture healing supported by the application of a PDGF-BB delivery device [2] in the rabbit Achilles tendon full transection model, we have analyzed the healing tendon tissue 3 weeks post laceration and compared it to a PDGF-BB free analogous tubular implant. We provide collagen I-to-III ratios for two different staining methods across different anatomical zones, including the native tendon, the similar to native tendon tissue, the core tissue, the interface tissue and the tissue inside the electrospun implant material.

## 3 Data Description

The data are stored as a Microsoft Excel file (Microsoft Corporation, Redmond, WA, USA) (.xlsx file) in two Mendeley Data repository services entitled Collagen I-to-III ratio data in healing tendon tissue: comparison of dual Herovici staining with two single immunohistochemical labelings for Collagen I and Collagen III (English version) - Mendeley Data and Spectral Analysis of Herovici stained sections of PDGF-BB DegraPol® or pure DegraPol® tube treated rabbit Achilles tendons and native tendons - Mendeley Data.

The excel file named Herovici and IHC Raw Data.xlsx is found in the first repository and includes the following sheets: (i) *HV-Col*; (ii) *Corr HV zu Col1;3*; (iii) *Corr NT+T-GF*; (iv) *Corr T* *+* *T-GF_NZ+SZ*; (v) *Corr T* *+* *T-GF_RZ*; (vi) *Corr T* *+* *T-GF_HZ*; (vii) *Corr T* *+* *T-GF_IS*; and (viii) *Calibration*, respectively. The excel file named Herovici tendons_ spectral analysis_2.xlsx is found in the second repository and includes two sheets: (i) *Spectra raw data in all NTs* and (ii) *Spectra raw data selected.*

### 3.1 Collagen I-to-III ratio data

**File** Herovici and IHC Raw Data_English.xlsx

This excel file is open access published in Mendeley Data Collagen I-to-III ratio data in healing tendon tissue: comparison of dual Herovici staining with two single immunohistochemical labelings for Collagen I and Collagen III (English version) - Mendeley Data and contains 8 sheets. In the first sheet ((i) *HV-Col*), column **A** refers to the file name; column **B** to the leg; column **C** to the internal lab code; column **D** to experimental group; column **E** to the rabbit colour (the rabbits were marked with a colour at their ears); column **F** to the side (either towards the muscle or towards the bone); column **I** to the field of view (FOV), column **J** to nothing (just left empty); column **G** to the distinct anatomical zone; column **H** to the staining method (either HV = Herovici or IHC immunohistochemical labeling); column **K** to Red signal intensity; column **L** to the Green signal intensity; column **M** to the Blue signal intensity; column **N** was left empty; column **O** to the Red-to-green ratio (calculated); column **P** to the Red-to-blue ratio (calculated); column **Q** was left empty; column **R** to the calculated mean of the Red-to-green ratio; column **S** to the calculated standard deviation (SD) of the Red-to-green ratio; column **T** was left empty; column **U** to the calculated mean of the Red-to-blue ratio; column **V** to the calculated standard deviation (SD) of the Red-to-blue ratio; column **W** was left empty; column **X** to the experimental group or therapeutic intervention (either with PDGF-BB = Tube+GF, without PDGF-BB = Tube only, or no treatment = NT); column **Y** was left empty; column **Z** calculated mean on the bone side; column **AA** calculated standard deviation (SD) on the bone side; column **AB** calculated mean on the muscle side; column **AC** calculated standard deviation (SD) on the muscle side; column **AG** staining method (HV or IHC); column **AH** experimental group; column **AI** calculated mean; column **AJ** calculated standard deviation (SD); column AK was left empty; column **AL** experimental group; column **AM** calculated mean; column **AN** calculated standard deviation (SD), column **AO** was left empty; column **AQ** to the calculated mean; column **AR** to the maximum value; column **AS** to the minimum value; and column **AT** to the calculated mean, respectively.

In the second (and the third to the seventh) sheet ((ii) *Corr HV zu Col1;3*), columns **A-H** are the same as in the first sheet, while columns **I-Q** were left empty. Columns R-Y were the same as in the first sheet. Column **Z** refers to the row number; column **AA** to the rabbit; column **AB** to the lab code; column **AC** to the anatomical zone, column **AD** to the side; column **AE** to the via IHC calculated collagen I-to-III ratio; column **AF** to the via HV calculated collagen I-to-III ratio. Then, columns **AG-AI** were left empty. In column **AJ** and the following columns, the correlation plots are shown: Plot 1 includes all three experimental group data; Plot 2 only NT and Tube only groups; and Plot 3 includes NT and Tube+GF groups, respectively.

In the fourth sheet ((iv) *Corr T* *+* *T-GF_NZ+SZ*), only the plots are different, i.e. in column AJ and the following the Plot 4 indicates data of the NZ and SZ zones for the groups Tube only and Tube+GF.

In the fifth sheet ((v) *Corr T* *+* *T-GF_RZ*), only the plots are different, i.e. in column AJ and the following the Plot 5 indicates data of the RZ zone for the groups Tube only and Tube+GF.

In the sixth sheet ((vi) *Corr T* *+* *T-GF_HZ*), only the plots are different, i.e. in column AJ and the following the Plot 6 indicates data of the HZ zone for the groups Tube only and Tube+GF.

In the seventh sheet ((vii) *Corr T* *+* *T-GF_IS*), only the plots are different, i.e. in column AJ and the following the Plot 7 indicates data of the IS zone for the groups Tube only and Tube+GF.

In the eighth sheet ((viii) *Calibration*), column **A** refers to the colour; columns **B-K** to the signal intensity value; column L was left empty; column **M** to the file name, column **N** to the lab code; column **O** to the colour, columns **P-AN** to the signal intensity value; column **AP** to the calculated mean and column **AQ** to the calculated standard deviation (SD).

**File**: Herovici tendons_ spectral analysis_2.xlsx

In this Excel file stored in the Mendeley repository Spectral Analysis of Herovici stained sections of PDGF-BB DegraPol® or pure DegraPol® tube treated rabbit Achilles tendons and native tendons - Mendeley Data there is a first sheet with the name *Spectra raw data in all NTs*. This sheet gives the names of specific fields of view (FOVs) in **line 4**, while in **line 5** the images of the corresponding FOVs are shown. In **lines 5–7**, the means of intensities of the colours Red, Green and Blue, respectively, are shown. Below in **line 8**, the histograms are provided. Then, the **lines 9** to **264** show the intensities for a specific colour (whole range 0–255 in column **C**) in column **D**. This structure is repeated in the following columns, i.e. the data in columns **F** and **G** are arranged in the same way as the data in columns **C** and **D**; etc. There is a second sheet called *Spectra raw data selected*. The sheet is exactly the same organized as the sheet *Spectra raw data in all NTs*.

## 4 Experimental Design, Materials and Methods

### 4.1 Herovici (HV) and immunohistochemistry (IHC)

**Fig. 1:**
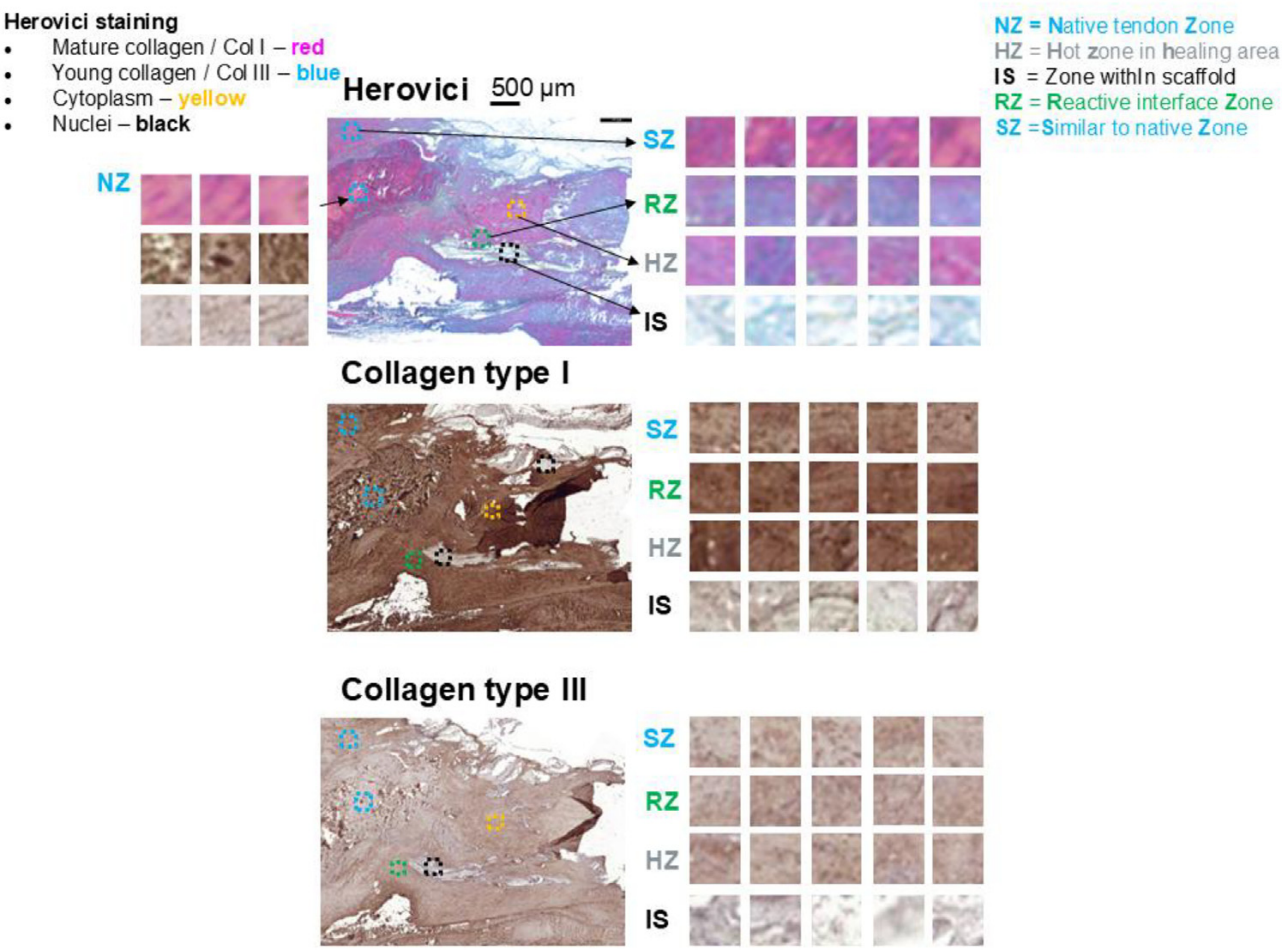
Three typical histological sections taken from the same side as serial sections, with five fields of view (FOVs) shown as squares on the right side. Top: Herovici dual staining with red for mature collagen (collagen type I) and blue for premature collagen (collagen type III). Middle: Immunohistochemistry for collagen type I with DAB staining (brown intensity is proportional to the collagen I content). Bottom: Immunohistochemistry for collagen type III with DAB staining (brown intensity is proportional to the collagen III content). In the right upper corner, the distinct anatomical zones are explained.

The Herovici staining and the immunohistochemical labelling for Collagen types I and III were performed for paraffin embedded histological sections as reported previously [1]. Information on colour attribution to Collagen I and III as well as cytoplasm and nuclei is given in Fig. 1. Explanation on which anatomical zone is found where in the tendon tissue is given for an example of a PDGF-BB treated rabbit Achilles tendon full transection model 3 weeks post-operation (Fig. 1), with distinct fields of view (FOVs) that are representative for the experimental groups.

## Limitations

While the *in vivo* experiments were limited to a three-week endpoint, longer observation periods would be highly valuable. This is particularly relevant given that the collagen I-to-III ratio undergoes time-dependent changes for up to twelve weeks post-surgery. In addition to the collagen I-to-III ratio across distinct anatomical zones, collagen fiber orientation and structural organization - specifically angular distribution and fibril diameter - represent critical further parameters that remained outside the scope of this study. Finally, cell density, spatial distribution, and the degree of vascularization were not assessed in the present study.

## Ethics Statement

The study was conducted in accordance with the veterinary office of the Canton Zurich, Switzerland, with the ethical approval under licence No. ZH 080/ 2021.

## CRediT Author Statement

**Gabriella Meier Bürgisser**: Conceptualization, Methodology, Data curation, Investigation, Writing – Original draft, Writing – Reviewing & Editing. **Olivera Evrova**: Methodology, Data curation, Formal analysis, Writing – Reviewing & Editing. **Pietro Giovanoli**: Conceptualization, Methodology, Supervision, Writing – Reviewing & Editing. **Maurizio Calcagni**: Conceptualization, Methodology, Supervision, Writing – Reviewing & Editing. **Johanna Buschmann**: Conceptualization, Supervision, Resources, Writing – Original draft, Writing – Reviewing and Editing, Funding acquisition, Project administration.

## Data Availability

Mendeley DataSpectral Analysis of Herovici stained sections of PDGF-BB DegraPol® or pure DegraPol® tube treated rabbit Achilles tendons and native tendons (Original data). Mendeley DataCollagen I-to-III ratio data in healing tendon tissue: comparison of dual Herovici staining with two single immunohistochemical labelings for Collagen I and Collagen III (English version) (Original data).
